# Mode matters: exploring how modes of cannabis administration affect THC plasma concentrations and subjective effects

**DOI:** 10.1186/s42238-025-00282-y

**Published:** 2025-05-23

**Authors:** Margy Y. Chen, Ashley Brooks-Russell, Angela D. Bryan, L. Cinnamon Bidwell

**Affiliations:** 1 Department of Psychology and Neuroscience, University of Colorado Boulder, 1905 Colorado Ave, Boulder, CO 80309 USA; 2 Injury and Violence Prevention Center, University of Colorado Anschutz Medical Campus, Aurora, CO USA; 3 Institute of Cognitive Science, University of Colorado Boulder, Boulder, CO USA

**Keywords:** Cannabis, Marijuana, Mode of administration, Route of administration, Subjective intoxication, High concentration cannabis

## Abstract

As cannabis products become increasingly accessible and novel modes of administration like dabbing and vaping gain popularity, understanding how these modes impact use experiences and abuse liability is crucial. Existing studies primarily utilized laboratory settings with lower-potency research-grade cannabis, failing to capture common modes among individuals who use higher-potency legal market products. This study investigated how modes of administration impact plasma tetrahydrocannabinol (THC) concentrations and subjective effects using naturalistic administration of legal market products. We conducted a secondary analysis of two quasi-experimental studies involving 252 participants (46.4% female). Participants completed a baseline assessment where they reported demographics, substance use, and medical history and an experimental assessment where they administered their products via their preferred modes. These modes were categorized into four general modes of use: dabbing, vaping, bong-like, and joint-like. Primary outcomes included plasma THC concentrations and subjective drug effects, both of which varied significantly across modes. Compared with modes typically associated with flower products (i.e., bong-, joint-like modes), dabbing was associated with higher plasma THC concentrations and subjective effects, indicating greater THC exposure when using this mode and its associated products. Interestingly, dabbing and vaping exhibited more rapid reductions in levels of subjective intoxication over time, suggesting that higher levels of intoxication may not be sustained for these modes. This study underscores the significant impact of modes of administration on THC exposure and subjective drug effects among individuals who regularly use legal market cannabis. Results demonstrate the need for future research to better characterize and account for modes of administration and their associated products.

## Background

Cannabis is the third most widely used substance in the United States after alcohol and tobacco products with over 42 million endorsing past-month use among people aged 12 or older (Substance 2022). The expanding legalization of medicinal and recreational cannabis has also led to a surge in the availability of methods for administering cannabis. These encompass smoking or inhalation (i.e., applying combustible heat), vaping or dabbing (i.e., typically applying non-combustible heat), topical application, as well as edible products such as cannabis-infused food or drinks (Borodovsky et al. 2016; Mahmoudinoodezh et al., 2022; Russell et al. 2018). Although knowledge about health and psychosocial correlates of cannabis use is rapidly growing, few studies have investigated routes or modes of administrations patterns and how they potentially impact plasma tetrahydrocannabinol (THC) levels and subjective effects. To our knowledge, studies on the effects of legal market cannabis products associated with modes of administration are scarce, largely due to cannabis’ status as a Schedule I substance under federal law (Mead and Mead 2017).

The three most prevalent methods of cannabis consumption are smoking, vaporizing, and ingesting through edibles (Borodovsky et al. 2016). There is some evidence from studies using research-grade cannabis products that different modes of administering the same product can have remarkably different effects. A previous crossover trial found that THC concentrations in whole blood and subjective intoxication levels were higher following vaping cannabis flower compared to smoking equal doses of flower through a handheld pipe (Spindle et al. 2018). Cooper and Haney showed that smoking joints (i.e., cannabis cigarettes rolled in papers typically made from hemp, rice, or other non-tobacco materials) was associated with higher plasma THC levels and greater subjective ratings of drug effects compared to smoking blunts (i.e., cannabis cigars wrapped in tobacco leaves) (Cooper et al. 2009)). In a self-report study with college students, students who used bongs reported more cannabis-related harms and dependence symptoms compared to those who primarily used joints (Swan et al. 2021). More recently, there is daily diary evidence that levels of subjective intoxication may vary as a function of mode of administration in daily life (Cloutier 2022). Compared to vape-only days, respondents felt more intoxicated on bong-only days, demonstrating mode-specific associations on daily use outcomes. Based on these pieces of evidence, it seems that there might be differences in drug effects and the potential risks linked to distinct modes of cannabis use. A limitation of existing data, however, is that none tested or queried the mode of vaporizing or dabbing, or the use of high-potency cannabis concentrates, which are becoming increasingly popular particularly in jurisdictions where cannabis has been legalized (Borodovsky et al. 2016; Borodovsky et al. 2017). Thus, more research is needed to understand whether and how various modes of administration (including more novel but increasingly popular modes of use) differentially impact the observed blood THC levels and subjective drug effects yielded by legal market cannabis.

Importantly, mode of use is often confounded with the cannabinoid concentration of the product being used. Smoking – historically the most common mode of cannabis administration – is limited to flower products, which typically contain 15–30% THC (Marijuana 2020). On the other hand, vaping is a mode that has become increasingly popular in recent years due to legislation legalizing cannabis use. There are a wide range of vaporizer options now available in the market (Budney et al. 2015; Lim et al. 2022). Larger devices previously used for vaporizing cannabis flower have been replaced by vape pens or other smaller electronic devices. These devices allow more discrete use and while these devices can be used for consumption of cannabis flower, they are more typically utilized for higher concentration products, such as oils, waxes, dabs, etc. (Budney et al. 2015; Schauer et al. 2016). Therefore, the effects of mode versus the cannabinoid concentration of product are difficult to disentangle.

Although existing pieces of evidence were suggestive, more research is needed to address the dearth of empirical data and fully characterize outcomes associated with contemporary modes of cannabis use (Russell et al. 2018; Cloutier 2022). Existing studies of acute effects of different modes of cannabis use have been conducted in controlled laboratory settings with lower THC concentration research-grade cannabis, and have not included increasingly popular forms of use (e.g., dabbing cannabis concentrate). Studies looking at the effects of legal market cannabis products used in a naturalistic manner are thus useful for examining blood THC levels and subjective drug effects of different commonly used modes of administration.

The current study extends prior research by offering comparisons between four general modes of use frequently observed among individuals who regularly consume cannabis recreationally, focusing on both plasma THC concentrations and subjective use-related outcomes. Given limited empirical data, the study approached the research question without predefined hypotheses. The analyses conducted were thus exploratory in nature, driven by the aim of unraveling novel insights with regards to the impacts of modes of administration on use experiences.

## Methods

The current study harmonized data from two quasi-experimental studies in Colorado where participants used retail market cannabis products in a naturalistic way (i.e., as they normally would). This approach allowed us to achieve sufficient variety of modes of administrations, enabling us to explore differences among four commonly used modes, with adequate sample size in each category. There were slight variations in the methods and measures, which are described in detail below separately as Study A (conducted at CU Boulder) and Study B (conducted at CU Anschutz). First, in Study B blood samples were collected to examine whole blood cannabinoid levels, which were subsequently converted to represent plasma levels, to align with Study A. Second, the first post-use assessment for Study A occurred acutely (i.e., as soon as possible, which was on average 14.59 min after use) while some participants in Study B completed their first post-use assessment at approximately 20 min since the start of use.^1^ Lastly, participants in Study B were first recruited into one of three groups based on cannabis use history: occasional (i.e., 3 or fewer times per week) use and smoking flower; daily use and smoking flower; and daily use and inhaling concentrate products. To align with the use profile of Study A, only those with daily use are included in the current analysis. Full methods for Study A and Study B were described in detail in three previously published papers.

### Study A

#### Participants and procedures

The research was approved by the University of Colorado Boulder Institutional Review Board. Participants were recruited as part of a series of randomized trials examining the acute effects of ad libitum cannabis use. Participants were recruited using social media postings and mailed flyers. Interested participants were screened over the phone by research staff. Criteria for inclusion were (a) age between 21 and 70; (b) used cannabis at least 4 times in the past month^2^; (c) endorsed prior use (at least once by self-report) of the highest potency of cannabis that could be assigned in the study (24% THC for flower cannabis or 90% THC for concentrates) with no adverse reaction; (d) no other non-prescription drug use in the past 60 days confirmed by urine toxicology screening; (e) no daily tobacco use; (f) breath alcohol level of 0 at screening and drinking 3 times or fewer per week and 5 drinks or fewer per occasion (men) and 4 drinks or fewer per occasion (women); (g) not pregnant (verified by urine pregnancy test) or trying to become pregnant; (h) not seeking treatment for drinking; (i) and not receiving treatment for psychotic disorder, bipolar disorder, or schizophrenia. Participants provided written informed consent and were compensated for participation during baseline ($50) and mobile laboratory appointments ($100).

##### Baseline appointment

Participants provided informed consent and completed questionnaires on demographics, substance use, and medical history. They then also underwent a blood draw to measure plasma cannabinoid concentrations and were randomly assigned to a potency condition and asked to purchase the assigned product at a local dispensary. Individuals using flowers were randomized to buy either 3 g of strain A (16% THC) or strain B (24% THC), both containing 1% cannabidiol (CBD). Individuals using concentrates were randomized to buy 1 g of concentrate A (70% THC, < 1% CBD), concentrate B (85% THC, < 1% CBD), or concentrate C (90% THC, < 1% CBD). Per State of Colorado law, all study products were labeled with their THC and CBD concentrations after testing in an International Organization of Standards 17025-accredited laboratory.

##### Mobile laboratory appointment

In order to comply with federal restrictions on state market cannabis use in a university setting, naturalistic cannabis use was assessed in our Mobile Pharmacology Laboratory (Bidwell et al. 2020; Drennan et al. 2021) during an appointment held approximately 5 days after the baseline appointment. Participants were asked to abstain from cannabis use on the day of their appointment. The visit began with pre-use assessments including self-report measures and a blood draw to verify abstinence. Participants then returned to their homes to use their assigned cannabis ad libitum through their preferred mode of administration. After using their products (average total time spent away from mobile laboratory, 14.59 min; range: 3–27 min), they returned to the mobile laboratory to complete the outcome measures while acutely intoxicated (acute post-use). They remained in the mobile lab until one hour after using and then completed the measures a final time (1-h post-use). Blood samples were collected at both post-use timepoints to verify adherence to condition assignment and to examine plasma cannabinoid levels. These data were analyzed and reported in two previously published studies (Bidwell et al. 2020; Gibson et al. 2022).

### Study B

#### Participants and procedures

The research was approved by the Colorado Multiple Institutional Review Board. Participants were recruited as part of a series of observational studies examining the acute effects of ad libitum cannabis use. Participants were recruited using social media postings, flyers, and word of mouth. Interested participants completed an online screening survey, and eligibility was confirmed over the phone by research staff. Criteria for inclusion were (a) age between 25 and 55; (b) used cannabis at least 2 times in the past month; (c) no other illicit drug use or mis-use of non-prescription drugs, confirmed by urine toxicology screening; (d) breath alcohol level of 0 at screening and drinking 3 times or fewer per week and 5 drinks or fewer per occasion (men) and 4 drinks or fewer per occasion (women); (e) not pregnant (verified by urine pregnancy test) or trying to become pregnant; (f) not seeking treatment for substance use; (g) and no untreated psychotic disorder, bipolar disorder, or schizophrenia. Participants provided written informed consent and were compensated for participation during baseline ($20) and in-person laboratory appointments ($280).

##### Baseline appointment

Participants provided informed consent and completed questionnaires on demographics, substance use, and medical history.

##### Laboratory appointment

To comply with federal restrictions on state market cannabis use in a university setting, naturalistic cannabis use was assessed in an off-campus laboratory. Participants were asked to abstain from cannabis use for at least 8 h for smoked products and at least 12 h for edible products before their laboratory visit. The visit began with pre-use assessments including self-report measures and a blood draw. Participants were asked to use their products (flower or concentrate) ad libitum for up to 15 min, using their preferred modes of administration (average consumption time: 7.26 min; range: 2–15 min). THC concentrations ranged from 15 to 33% for flower products and from 60 to 90% for concentrate products, while all products contained less than 2% CBD. After using their products, they completed the outcome measures acutely and 60 min after use. Blood samples were collected to examine whole blood cannabinoid levels, which were subsequently converted to represent plasma levels, to align with Study A. Subsets of these data were analyzed and reported in two previously published studies (Henthorn 2022; Limbacher 2024).

#### Measures

##### Mode of administration

In Study A, participants were asked to report their modes used to consume cannabis with the following question: “What method did you use to consume the flower/concentrate?” In Study B, research staff observed and recorded the mode of use for each participant during their laboratory visit. Responses included bong, bowl, pipe, bubbler, joint, blunt, spliff, dab rig, dab tube, vape pen, hash pen, and vaporizer. In both studies, modes of use were collapsed based on similarity into four groups: Bong-like, Joint-like, Dabbing, and Vaping. Notably, all bong and joint methods were only reported by participants using cannabis flower, while all dabbing and vaping methods were only reported by participants using cannabis concentrates. Thus, although vaporizers can be used to consume both cannabis flowers and concentrates, participants who endorsed vaping in the current study only vaped concentrates.

##### Plasma THC concentrations

In Study A, a certified phlebotomist collected ~ 50 mL of venous blood through venipuncture of a peripheral arm vein using standard, sterile phlebotomy techniques, which was stored on ice in the mobile laboratory. Upon return to the laboratory, plasma was separated from erythrocytes by centrifugation at 1000xg for 10 min, transferred to a fresh microcentrifuge tube for phytocannabinoid analysis and a separate microcentrifuge tube for endocannabinoid analysis, and stored at −80°C. Plasma samples were sent to the iC42 Lab at the University of Colorado Anschutz Medical Campus. THC concentration was quantified using validated high-performance liquid chromatography/mass-spectroscopy (API5500) (Klawitter et al. 2017). In Study B, a certified phlebotomist collected 5 ml of venous blood through venipuncture of a peripheral arm vein using standard, sterile phlebotomy techniques. Samples were placed on a rocker immediately after collection and rocked for mixing for 5 min until freezer storage at−16º C. Samples were transferred to the laboratory in a dry ice cooled carrier for analysis at Colorado State University within three weeks of collection. THC concentration was quantified using validated LC–MS/MS analysis (an Agilent 1290 UHPLC coupled to an Agilent 6460 triple quadruple mass spectrometer equipped with an Agilent Jet Stream electrospray ionization source (Agilent, Santa Clara, CA). To harmonize data across studies, whole blood THC concentrations collected in Study B were first converted to plasma THC concentrations with 1.7 ratio as plasma levels of THC was collected in Study A. This conversion is based on prior research, which has consistently demonstrated this plasma-to-blood ratio (Raikos et al. 2014; Skopp et al. 2002).

##### Measures of subjective drug effect

Both studies utilized the Addiction Research Center Inventory–Marijuana (ARCI-M) (Haertzen 1987). The 12-item ARCI scale was used to measure subjective cannabis intoxication. Participants were asked to indicate if they had experienced the described effect (e.g., “Things around me seem more pleasing than usual”) in a true–false format (α = 0.75). One point was given to each true response and sum scores were computed. Greater sum scores indicated higher levels of subjective intoxication.

Both studies also employed the Drug Effect Questionnaire (DEQ) (Fraser et al. 1961). The 5-item DEQ was used to assess acute subjective effects of cannabis use (e.g., “Do you like any of the effects you are feeling right now?”) on a 5-point Likert-type scale with responses ranging from 1 “Not at all” to 5 “Extremely.” Responses were summed to compute a scale score (α = 0.81).

Additionally, two items from the DEQ (“Do you feel a drug effect right now?”, “Are you high right now?”) that capture subjective intoxication specifically were analyzed separately (Morean et al. 2013). These two items were highly correlated with one another at both acute (*r* = 0.78) and 1-h post-use (*r* = 0.87) and thus were combined to form a two-item subjective intoxication scale.

### Analyses

Repeated measures analyses of variance (ANOVA) were conducted with a linear mixed-modeling framework estimating random intercepts for participants to compare THC levels and subjective outcome variables (i.e., ARCI and DEQ) across the four modes of administration groups. The model for plasma THC levels included time (both linear and quadratic effects of time across three time points: pre-use, acute post-, and 1-h post-use^3^), mode of administration, and the time by mode interaction. The same predictors were included in the models for subjective outcome variables except that time was contrast coded as acute post-use vs. 1-h post-use (Bidwell et al. 2020). In cases of a significant time by mode interaction, post hoc pairwise comparisons with Bonferroni correction at each time point were conducted for all four modes of administration. All baseline characteristics, as well as consumption time and potency of the products, were explored as covariates for each outcome but did not change the pattern of findings. Thus, results obtained from simpler models were presented. All analyses were conducted in R version 4.3.2 (http://www.posit.co) using the lme4 package version 1.1–35.1 (Bates et al. 2015), which implements Maximum Likelihood (ML) estimation.

## Results

### Demographics and between-study differences

One hundred and fifty-nine participants (females = 76, males = 83) were recruited for Study A while ninety-three participants (females = 41, males = 49, non-binary individuals = 3) were recruited for Study B. Thus, the final sample consisted of 252 participants (females = 117, males = 132, non-binary individuals = 3). Table 1 provides baseline characteristics of participants across the two studies.

**Table 1:** Study comparison on demographics and baseline characteristics

	Study A (*n* = 159)	Study B (*n* = 93)	Analysis
Demographics			
			*t*
Age	30.20 (7.92)	32.55 (6.32)	−2.56*
BMI	24.97 (3.52)	26.21 (4.99)	−2.12*
			*χ2*
Gender (% female)	47.9%	44.1%	4.42
Race/ethnicity			1.06
American Indian or Alaska Native	2.1%	0%	
Asian	7.7%	1.1%	
Black or African American	5.6%	10.8%	
White	68.5%	67.7%	
Hispanic or Latino	4.9%	14%	
Native Hawaiian and Pacific Islander	0.7%	0%	
More than one race	6.3%	5.4%	
Employment (% full-time employed)	48.3%	88.2%	13.85**
Education (% Bachelor or higher)	44.1%	66.7%	14.22*
			*t*
Substance use history and current use			
Age of onset of regular cannabis use	18.49 (*6.26*)	19.40 (*5.13*)	−1.24
Days of cannabis use in past 30 days	23.73 (*3.63*)	29.47 (*2.57*)	−5.29*
Number of drinks consumed per typical drinking day	2.05 (*1.04*)	3.82 (*3.76*)	−4.39***
			*t*
Cannabis use during *ad libitum* administration			
Consumption time / time spent away from mobile lab	14.59 (*6.66*)	7.26 (*4.15*)	10.56***
%THC concentrations of products	52.52 (*5.71*)	51.10 (*6.85*)	1.72
Mode of administration (%)			78.96***
Dabbing	105 (*66.0*)	21 (*22.6*)	
Vaping	23 (*14.5*)	22 (*23.7*)	
Joint-like modes	10 (*7.0*)	21 (*22.6*)	
Bong-like modes	21 (*14.7*)	29 (*31.2*)	

*p < .05, **p < .01, ***p < .001

### Plasma THC concentrations

**Fig. 1:**
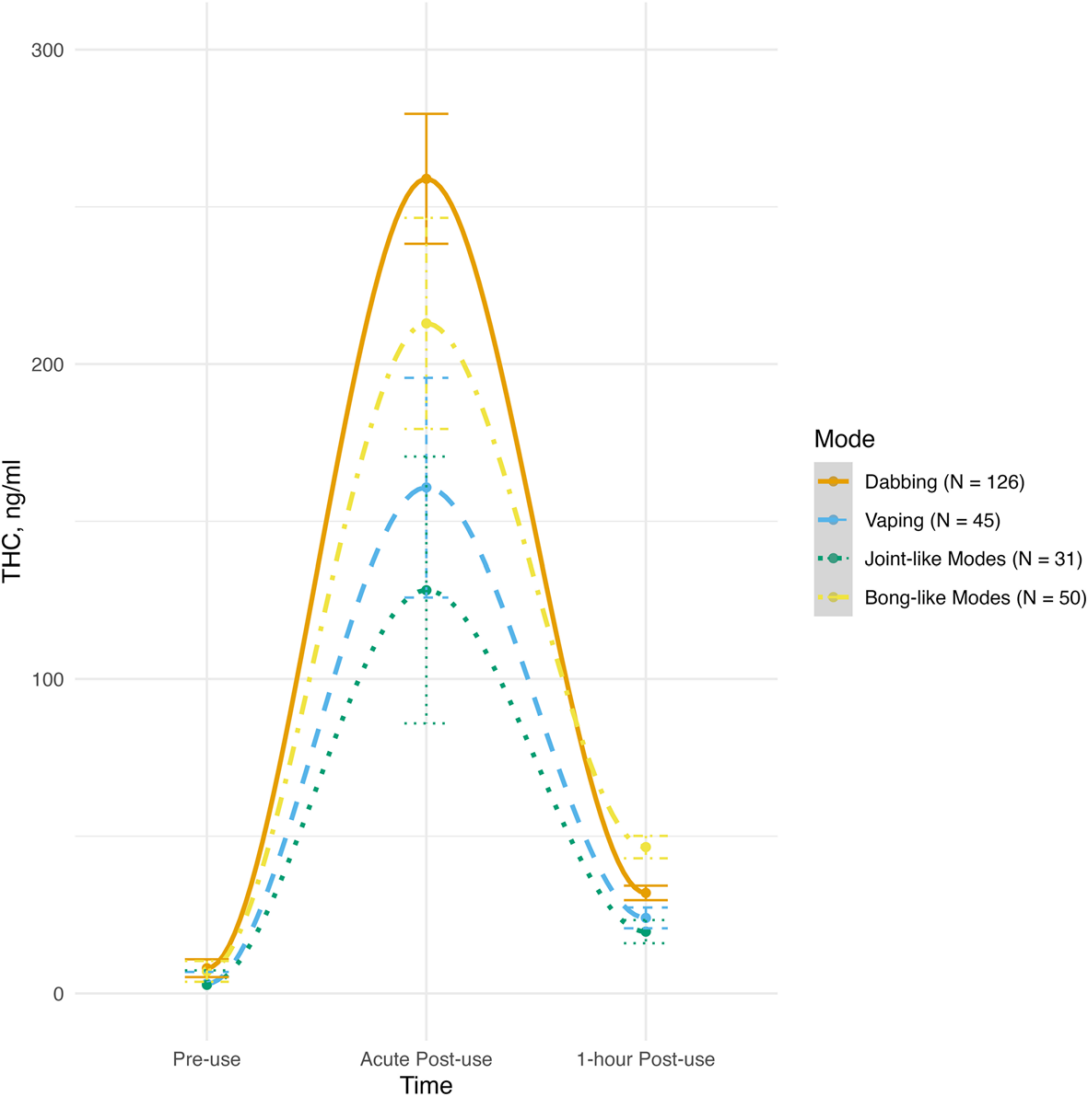
Plasma THC levels by mode of administration

There was a significant quadratic effect of time for plasma THC concentrations, such that levels peaked at acute post-use and then dropped (*F(*1, 247) = 41.71, *p* < 0.001). There was also a significant quadratic time X mode interaction (*F(*1, 247) = 2.55, *p* = 0.045). Thus, participants’ plasma THC levels varied significantly based on their modes of administration. Post-hoc pairwise comparisons revealed that at acute post-use, dabbing was associated with significantly higher plasma THC levels than joint-like modes (*t* = 2.77, *p* = 0.034). Dabbing was also associated with higher plasma THC levels acutely post-use than vaping, but this difference did not reach traditional levels of statistical significance (*t* = 2.42, *p* = 0.079). Further, plasma THC levels did not significantly differ between dabbing and bong-like modes acutely post-use (*t* = 1.77, *p* = 0.258). No significant differences were found at 1-h post-use. See Fig. 1 for plasma THC levels across the three assessment time points for each mode of administration.

### ARCI-M

**Fig. 2:**
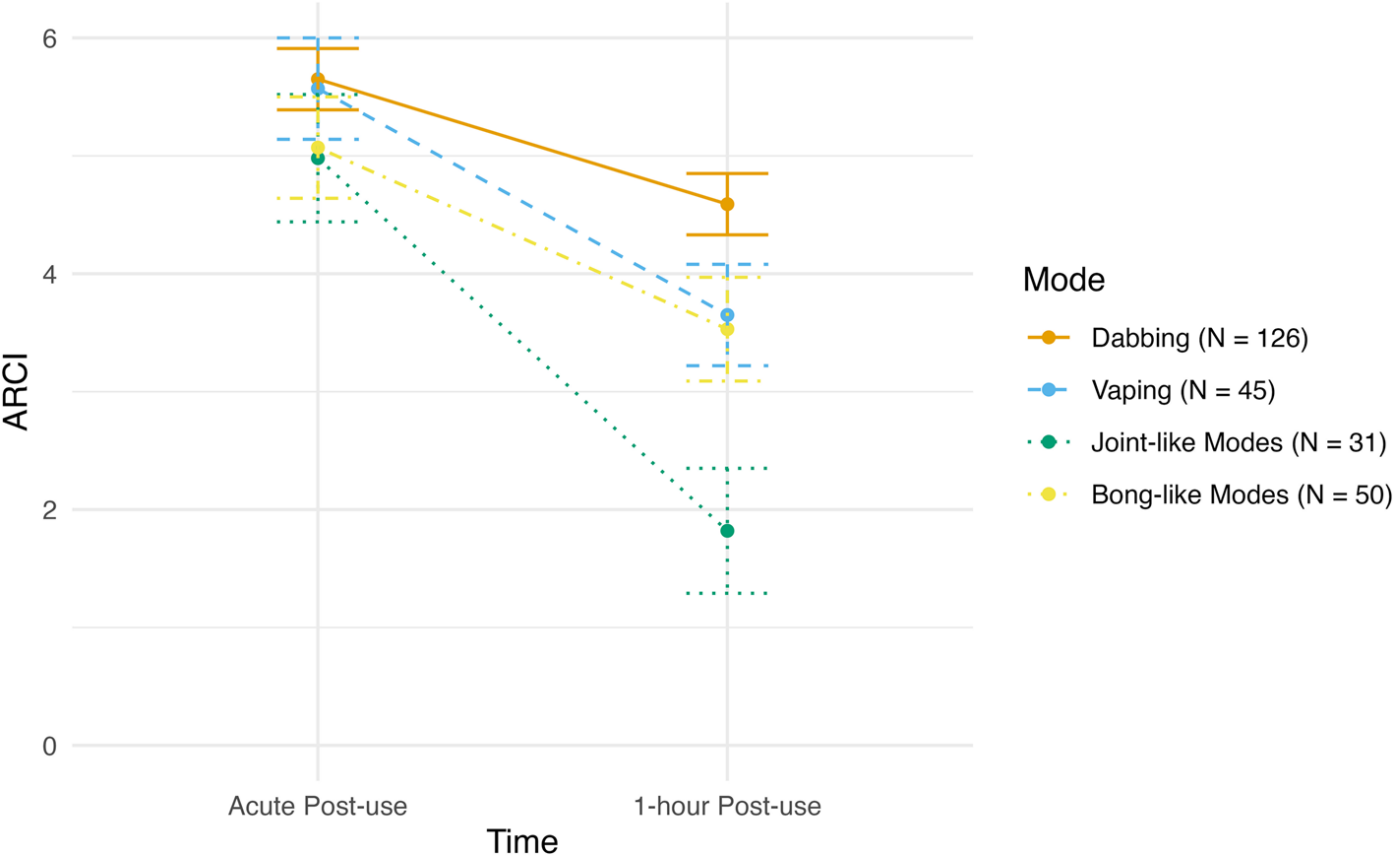
Changes in the ARCI marijuana scale by mode of administration. The Addiction Research Center Inventory (ARCI) is a self-report questionnaire designed to assess whether respondents are currently experiencing the effects of psychoactive drugs

There was a negative linear effect of time for ARCI-M scores (*F*(1, 249) = 60.21, *p* < 0.001), as across all modes of administration greater subjective effects were found at acute post-use and reduced subsequently. There was also a marginal main effect of mode (*F*(1, 249) = 2.55, *p* = 0.057) as well as a significant time X mode interaction (*F*(1, 249) = 13.12, *p* < 0.001). Post-hoc pairwise comparisons revealed that at acute post-use, dabbing was associated with stronger subjective effects (i.e., greater ARCI scores) than bong-like modes (*t* = 2.90, *p* = 0.020), joint-like modes (*t* = 5.30, *p* < 0.001), and vaping (*t* = 2.67, *p* = 0.032). Additionally at acute post-use, bong-like modes (*t* = 2.48, *p* = 0.032) and vaping (*t* = 2.67, *p* = 0.032) were associated with stronger effects than joint-like modes. Dabbing was still associated with stronger subjective effects than bong-like modes (*t* = 2.97, *p* = 0.012), joint-like modes (*t* = 5.43, *p* < 0.001), and vaping (*t* = 2.90, *p* = 0.005) at 1-h post-use. Also at 1-h post-use, bong-like modes (*t* = 2.55, *p* = 0.032) and vaping (*t* = 2.43, *p* = 0.039) were associated with significantly stronger subjective effects than joint-like modes. See Fig. 2 for ARCI scores across two post-use time points for each mode of administration.

### DEQ

**Fig. 3:**
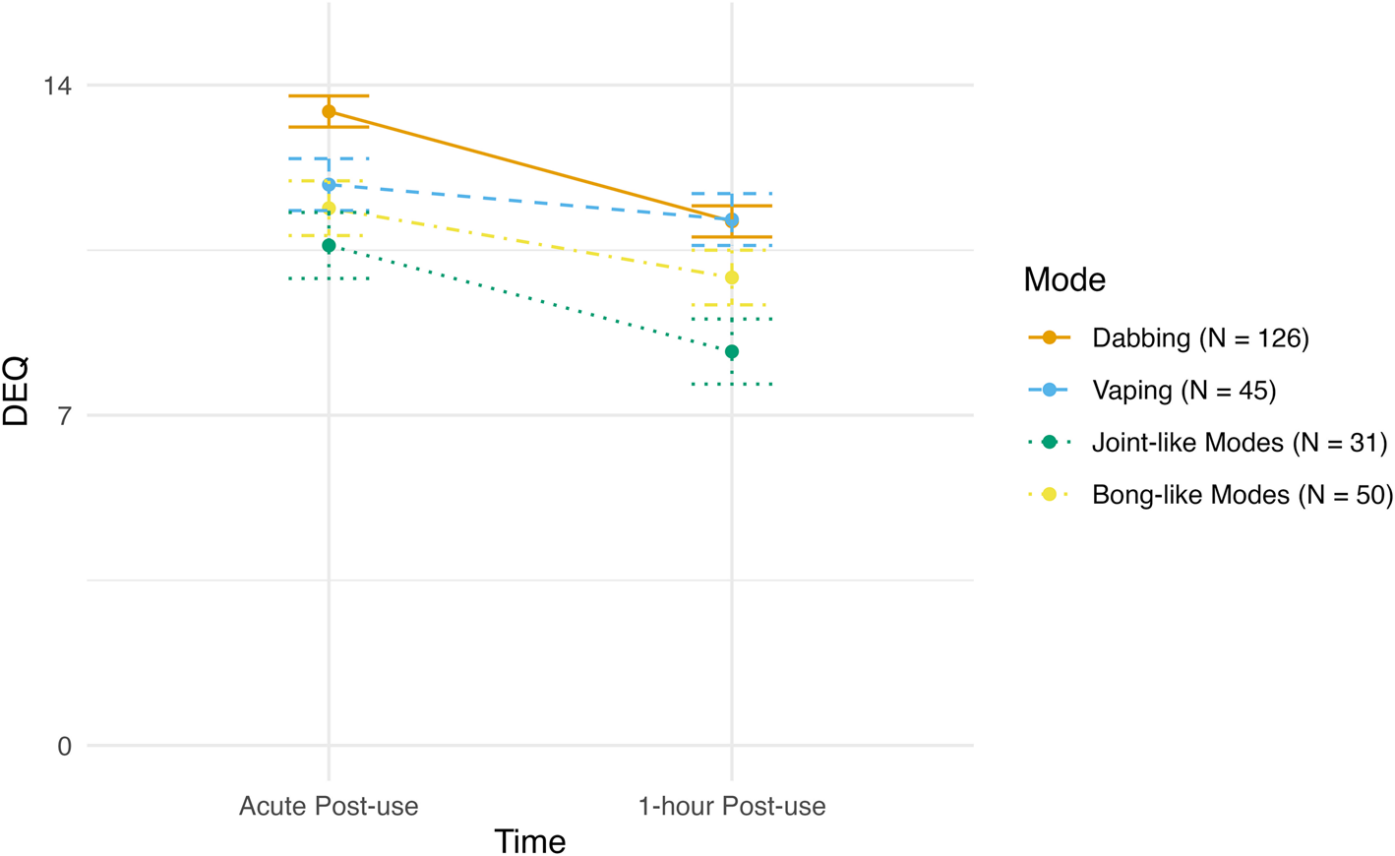
Changes in the DEQ by mode of administration. The Drug Effects Questionnaire (DEQ) is a self-report questionnaire widely used to assess respondents’ subjective experiences of psychoactive drugs

There was a negative linear effect of time for DEQ, as across all modes participants reported greater drug effects at acute post-use, which then reduced over time (*F*(1, 249) = 68.43, *p* < 0.001). There was also a significant main effect of mode of administration (*F*(1, 249) = 6.42, *p* = 0.003) while the time X mode interaction was not significant (*F*(1, 249) = 1.61, *p* = 0.187). Averaging across the two time points, dabbing was associated with significantly greater drug effects than vaping (*t* = 2.09, *p* = 0.038), joint-like modes (*t* = 3.91, *p* < 0.001), and bong-like modes (*t* = 2.59, *p* = 0.010). See Fig. 3 for subjective drug effects across two post-use time points for each mode of administration.

### Subjective intoxication

**Fig. 4:**
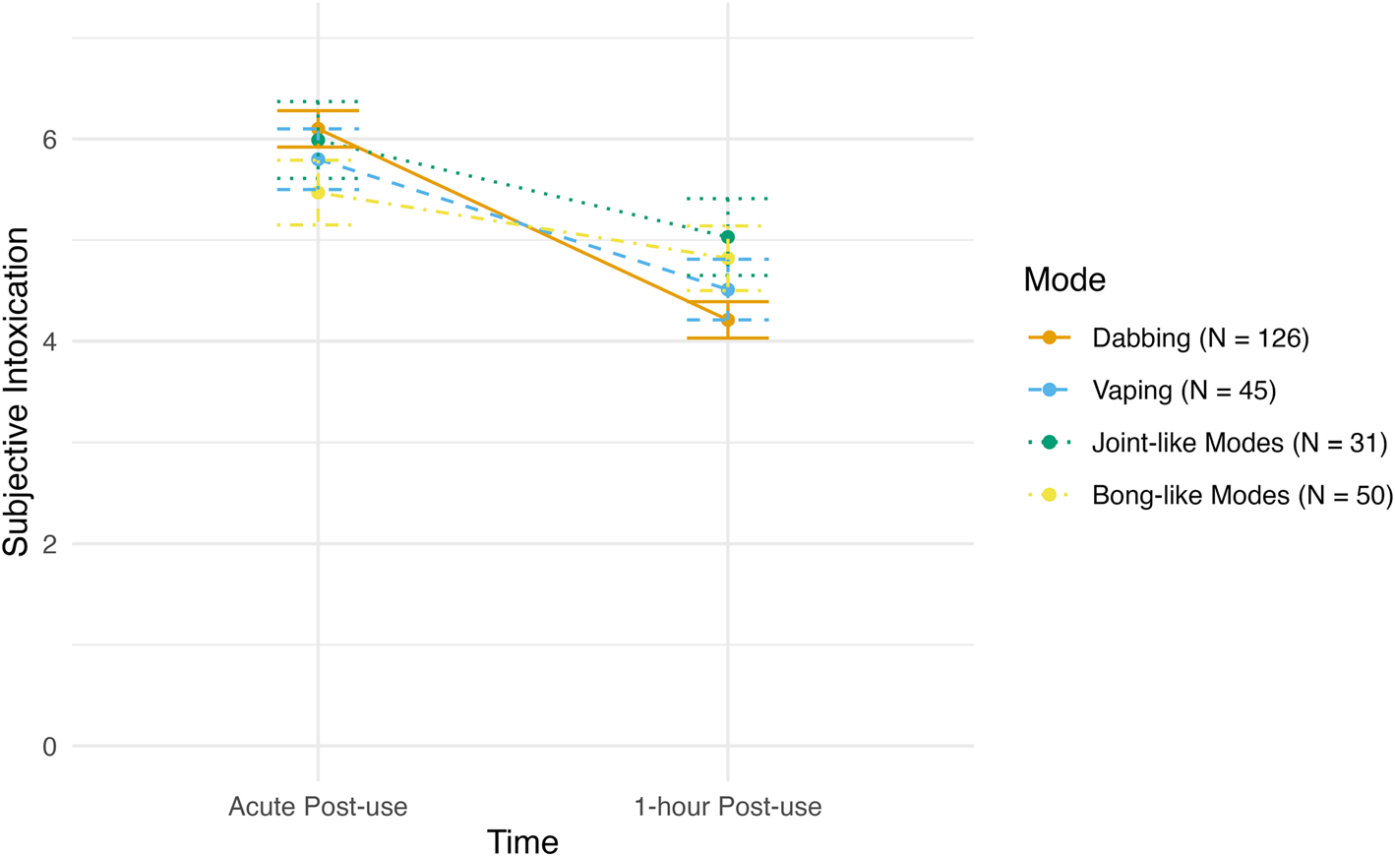
Changes in subjective intoxication by mode of administration. Two items in the DEQ (“Do you feel a drug effect right now?”, “Are you high right now?”) are used to form a subjective intoxication subscale

There was a negative linear effect of time with levels of subjective intoxication, as across all modes higher levels of subjective intoxication were reported at acute post-use and reduced over time (*F*(1, 249) = 21.78, *p* < 0.001). There was also a main effect of mode of administration (*F*(1, 249) = 4.73, *p* = 0.003) as well as a significant time X mode interaction (*F*(1, 249) = 4.53, *p* = 0.004). Post-hoc pairwise comparisons demonstrated that at acute post-use, dabbing was associated with higher levels of subjective intoxication than bong-like modes (*t* = 4.51, *p* = 0.032) and vaping (*t* = 2.70, *p* = 0.022). Additionally at acute post-use, participants who endorsed joint-like modes reported significantly higher subjective intoxication than participants who endorsed bong-like modes (*t* = 2.94, *p* = 0.021). At 1-h post-use, dabbing was associated with lower levels of subjective intoxication than bong-like modes (*t* = −3.13, *p* = 0.012) and joint-like modes (*t* = −2.63, *p* = 0.044). See Fig. 4 for levels of subjective intoxication across two post-use time points for each mode of administration.

## Discussion

The current analysis provides a thorough evaluation of the acute effects associated with four modes of administering legal market cannabis products containing THC among participants who regularly use cannabis. Findings suggest that both plasma THC levels and subjective drug effects differed significantly based on common real-world modes of administration. Consistent with our prior finding that concentrate use was associated with higher plasma THC levels than flower use, while exhibiting similar levels of short-term impairment and intoxication (Bidwell et al. 2020), participants who administered cannabis via dabbing in the current study also showed higher plasma THC levels immediately post-use than participants using other modes. However, despite higher THC exposure, they did not consistently report significantly greater subjective drug effects, which is consistent with prior literature (Bidwell et al. 2020) and could be due to individual differences in tolerance and/or THC metabolism. Notably, dabbing was linked to greater ARCI and DEQ acutely and one-hour post-use, indicating greater perceived physiological and psychological effects. Among the other three modes, vaping was associated with greater ARCI scores, indicating greater short-term impairment. Interestingly, findings on the 2-item subjective intoxication scale exhibited a different pattern. Although dabbing and vaping were associated with higher levels of subjective intoxication immediately after use, these two modes showed more rapid reductions at 1-h post-use compared to the other two smoking modes (i.e., bong- and joint-like modes). This suggested that while dabbing and vaping might be related to greater intoxication immediately after use, the effects might wear off more quickly compared to the other two modes, potentially leading to more frequent use to maintain the desired level of intoxication. This pattern of use could be a risk factor for problematic cannabis use, as the need for quicker reuptake may encourage higher overall consumption.

Moreover, compared to the other modes of administering cannabis, dabbing on average produced relatively higher plasma THC levels and greater subjective effects acutely. Thus, compared to traditional methods of smoking flower cannabis (e.g., joints or bongs) and vaporized cannabis, dabbing appeared to be associated with greater THC exposure. This is likely because dabbing involves using products with higher THC concentrations (e.g., 60–90% THC) whereas smoking uses flower products typically containing 15–25% THC (Marijuana 2020). Additionally, traditional smoking may result in greater THC loss due to pyrolysis or combustion at more elevated temperatures and producing sidestream smoke (Pomahacova et al. 2009). However, contrary to prior findings, vaporized cannabis did not consistently produce significantly higher blood THC concentrations than smoked cannabis (Spindle et al. 2018). In the present study, participants who endorsed bong-like modes on average had higher plasma THC levels than those who endorsed vaping despite vaping involving the use of higher-THC concentration products, although this difference did not reach significance. It is important to note that the previous study sampled individuals who use cannabis infrequently and the cannabis administered contained substantially lower THC concentrations than cannabis products typically available on the legal market (Spindle et al. 2018), which were used in the current study. Moreover, their participants vaped flower products whereas in the current study participants only vaped (concentrate) oils. Further, the discrepancy in results could also be due to procedural differences, as the current study allowed ad libitum use and enabled participants to self-titrate their THC doses and desired high.

While previous researchers have employed controlled laboratory designs, this study is the first, to our knowledge, to compare common modes of cannabis administration in plasma THC levels and subjective drug effects using naturalistic administration of legal market cannabis products among individuals who use cannabis regularly. Additionally, this study represents a crucial initial step in investigating the role of modes of administration in determining use experiences and outcomes. Findings highlight the importance of acknowledging that certain modes of administering cannabis, such as dabbing and vaping concentrates, may lead to more pronounced drug effects compared to others. While individuals with more experience with using cannabis may already be aware of these differences in intoxication levels, these results provide empirical support that can guide consumers, particularly those with less experience or less familiar with different administration methods. As the legal cannabis landscape continues to expand, future research should delve deeper into the effects of other novel methods of cannabis administration and include individuals with diverse levels of experience with using cannabis, given the potential variations in pharmacokinetic and pharmacodynamic responses across different products and consumption patterns.

While our findings are both novel and significant, they must be considered in the context of the following methodological limitations. First, our sample consisted predominately of individuals who use cannabis regularly or daily. They were also mostly white adults with a college education, thus limiting the generalizability of our findings. Given that participants use cannabis regularly or daily, their prior experiences using different products with various modes of administration may have influenced the effects they experienced in the current study. Further, our participants likely had higher tolerance than those with different use patterns and thus may self-select into their preferred products with higher THC concentrations such as concentrates or oils. Future studies should investigate the role of mode of administration in a more diverse sample including individuals who are new to using cannabis and those who use infrequently. Moreover, despite the strengths of using naturalistic administration procedures, ethical constraints made random assignment of participants to different modes of administration impossible. And although participants from the two studies were matched in terms of use frequency, there could be pre-existing differences between the two samples that were not accounted for. Lastly, participants’ reported modes of administration were collapsed into four general categories. Although this allowed comparisons between four general modes of administering cannabis popular among consumers, specific characteristics about consumers’ modes or devices (e.g., vaporizers with varying levels of power output and temperatures) should be explored further as they may influence THC exposure and experienced drug effects.

In conclusion, the present study provides novel data to demonstrate that modes of administration likely play a role in THC exposure and experienced drug effects of cannabis reported by individuals who consume cannabis regularly using commercially available cannabis products. Future research is needed to better characterize and account for modes of cannabis administration to inform safe uses as mode or route patterns quickly change over time among individuals using legal market cannabis products.

## Data Availability

The datasets used and/or analyzed during the current study are available from the corresponding author on reasonable request.

## Abbreviations

THC: Tetrahydrocannabinol
CBD: Cannabidiol
LC–MS/MS: Liquid chromatography-tandem mass spectrometry
ARCI-M: Addiction Research Center Inventory –Marijuana
ARCI: Addiction Research Center Inventory
DEQ: Drug Effects Questionnaire
ANOVA: Analysis of Variance

## Footnotes

1 Although some participants in Study B (*n* = 64, consumption time = 6.33) completed their first post-use assessment approximately 20 min since the start of use while others (*n* = 29, consumption time = 7.86) completed their first post-use assessment as soon as possible, consumption time did not statistically differ (*t* = -1.02, *p* = 0.09).

2 Initial recruitment for Study B was limited to participants who reported using cannabis at least 2 times in the past month. Due to this discrepancy between the two studies, inclusion in data analysis was further limited to individuals who use cannabis daily in Study B.

3 Some of the participants in Study B completed their first post-use assessment at about 20 min after use while others were assessed acutely. To control for potential variance caused by this, consumption time was included as a covariate for each outcome in additional analyses, but results did not differ from models without it as a predictor. Thus, results obtained from simpler models were presented.
